# Genetic Counseling for Autism Spectrum Disorder in an Evolving Theoretical Landscape

**DOI:** 10.1007/s40142-016-0099-9

**Published:** 2016-06-24

**Authors:** Brenda Finucane, Scott M. Myers

**Affiliations:** Autism & Developmental Medicine Institute, Geisinger Health System, 120 Hamm Drive, Ste 2A, Lewisburg, PA 17837 USA

**Keywords:** Autism spectrum disorder, Genetic counseling, Schizophrenia, Intellectual disability, Copy number, Pathogenic variant, Chromosomal microarray, Whole exome sequencing

## Abstract

**Purpose of Review:**

Psychiatry is steadily moving toward a new conceptualization of brain disorders that blurs long-held diagnostic distinctions among neurodevelopmental and psychiatric conditions, including autism. Genomic discoveries are driving these changing perceptions, yet there has so far been minimal impact on traditional genetic counseling practices that continue to view autism through the lens of a dichotomous, all-or-none risk model.

**Recent Findings:**

High rates of comorbidity exist across autism spectrum disorder, schizophrenia, intellectual disability, and other brain-based disorders. Recent epidemiological studies have shown that co-occurrence of neurodevelopmental and psychiatric disorders is the rule, rather than the exception, in affected individuals and within families. Moreover, studies of chromosomal microarray analysis and whole exome sequencing have now detected many of the same pathogenic copy number and sequence-level variants across cohorts with different clinical presentations.

**Summary:**

Going forward, the genetic counseling field will need to significantly adapt its approaches to pedigree interpretation, variant analysis, and patient education to more precisely describe both the chance and the nature of autism recurrence in terms of a continuum of brain dysfunction. These efforts will have implications for multiple practice areas and require philosophical changes for experienced practitioners and for the training of new genetic counselors. Resetting entrenched dichotomous notions about autism and other brain-based manifestations of genetic conditions will require a strategic educational effort on the part of the genetic counseling profession.

## Introduction

When first described as a childhood-onset psychiatric condition over 70 years ago, autism was considered a rare disorder of unknown etiology. Leo Kanner originally reported on 11 children with communication disturbances, a relative lack of motivation for social and emotional interaction, and preference for an excessive degree of routine and “sameness” in their environments [2]. Over the next few decades, autism was widely regarded as a type of childhood schizophrenia (SCZ), and some theorized that it was a form of psychotic withdrawal in response to an emotionally cold and distant mother [3•]. By the 1970s, a more enlightened understanding of autism as a biologically based condition began to emerge. Recent decades have seen the refinement of diagnostic criteria for autism, as well as the parsing out and later recombining of autism subtypes under the current umbrella of autism spectrum disorder (ASD) [3•, 4, 5].

ASD is a complex, chronic neurodevelopmental disorder that is clinically and etiologically heterogeneous, yet highly heritable, with a recent meta-analysis of twin studies suggesting heritability estimates from 64 to 91 % [6]. The genetic architecture of ASD includes rare pathogenic copy number variants (CNVs) and single nucleotide variants (SNVs), in addition to inherited background polygenic risk [7–9]. By a ratio of ~4.5 to 1, boys are more commonly affected by ASD than girls, leading to speculation about female protective effects as well as genetically conferred male vulnerabilities [10–14, 15•].

The much publicized increase in ASD prevalence over the past three decades has been well-documented in public health surveillance studies, with the most recent large-scale report showing a steady rate of ASD of 1 in 68 children in the US between 2010 and 2012 [16]. The extent to which there has been a true increase in ASD incidence, versus the collective effects of diagnostic and ascertainment changes, continues to be debated [17–20]. There is substantial evidence that non-etiological factors, such as “diagnostic substitution” [i.e., intellectual disability (ID) diagnoses decreased at the same time ASD diagnoses increased], have played a role. However, not all of the “autism epidemic” can be explained by these factors, and research efforts continue to investigate genetic, environmental, and other potential contributors [17–22].

Deliberations about clinical diagnostic criteria for ASD have largely played out within the fields of pediatric psychiatry and psychology, with relatively little cross-fertilization from research efforts to uncover its biological basis. Recent genomic evidence has shed new light on the interconnectedness of many brain-based pediatric and adult-onset psychiatric conditions, including ASD, ID, and SCZ, at the same time revealing biological inconsistencies with clinical diagnoses defined by psychiatry’s Diagnostic and Statistical Manual of Mental Disorders (DSM) [4]. The discovery of linked genetic underpinnings among several DSM-based conditions has led to emerging new perspectives on diagnosis, interventions, and research strategies for ASD [23, 24•, 25•]. This evolving conceptualization of autism as one constellation of symptoms within a larger universe of interconnected brain dysfunction has the potential to radically change genetic counseling for neurodevelopmental and psychiatric disorders.

## Autism as Developmental Brain Dysfunction (DBD)

Recently, the long-standing practice of defining ASD and other psychiatric diagnoses based on dichotomous, all-or-none symptom constellations (e.g., autism vs. no autism) has been called into question [23, 24•, 26]. Some individuals exhibit behavioral features of autism, for example, without fully meeting the criteria for an ASD diagnosis. Moreover, high rates of comorbidity exist across ASD, SCZ, ID, and other brain disorders; and epidemiological studies have shown that co-occurrence of neurodevelopmental and psychiatric disorders is the rule, rather than the exception, in affected individuals and within families. ASD, ID, SCZ, attention deficit/hyperactivity disorder (ADHD), epilepsy (EP), and bipolar disorder (BPD), for example, have long been conceptualized as clinically distinct entities but have overlapping symptoms, high rates of co-occurrence, etiologic heterogeneity, and shared risk factors; sometimes different disorders cluster within the same families [24•]. It has become increasingly clear that the genome does not respect psychiatry’s clinical diagnostic boundaries; chromosomal microarray analysis (CMA) and whole exome sequencing (WES) studies have now detected many of the same pathogenic CNVs and SNVs across cohorts with different clinical presentations, as illustrated in Table 1 [27–36]. It has been proposed that neurodevelopmental and psychiatric diagnoses should not be viewed as causally and pathophysiologically distinct, but as the consequences of DBD, a common denominator that reflects altered neural development which can manifest clinically in diverse ways [24•, 37].

**Table 1:** Variable expressivity of the pathogenic recurrent copy number variants and single nucleotide variants most commonly identified in ASD cohorts

	ASD	ID/DD	EP	SCZ
CNV				
16p11.2 deletion	X	X	X	
16p11.2 duplication	X	X	X	X
15q11.2-q13.1 (BP2–BP3) duplication	X	X	X	X
15q13.2-q13.3 (BP4–BP5) deletion	X	X	X	X
1q21.1 duplication	X	X	X	X
22q11.2 duplication	X	X	X	
16p13.11 deletion	X	X	X	X
7q11.23 duplication	X	X		X
16p12.2 deletion	X	X	X	X
17q12 deletion	X	X	X	X
SNV				
*NRXN1*	X	X	X	X
*CTNNA3*	X	X	X	X
*CHD8*	X	X	X	X
*SCN2A*	X	X	X	
*ADNP*	X	X	X	
*PTEN*	X	X	X	
*SCN1A*	X	X	X	
*SHANK3*	X	X	X	X
*DYRK1A*	X	X	X	
*SYNGAP1*	X	X	X	X

References [24•, 28, 33–36]

DBD represents a group of developmental, neurological, and psychiatric conditions characterized by cognitive, behavioral, language, motor, and other brain-based functional impairments [24•]. Rather than dichotomous, all-or-none disorders, ASD, ID, ADHD, SCZ, and other DBD are now thought to reflect varying degrees of dysfunction along a broad continuum of measurable (quantitative) human traits, including intelligence, social responsiveness, attention, language abilities, motor skills, and imaginative thought. All humans fall somewhere along the continuum of function for these quantitative traits, with diagnoses such as ASD representing the extreme end of a spectrum where function is impaired to the degree that it warrants a clinical label. Current opinions from top researchers in the field and by the US National Institutes of Mental Health suggest that ASD and other DBD can best be studied and conceptualized through quantitative behavioral and cognitive research, irrespective of artificially defined clinical diagnostic boundaries [23, 24•]. An important corollary is that DBD candidate gene discovery can be maximized by combining datasets from different neurodevelopmental and psychiatric disorders [28]. This changing conceptualization of autism and its interconnectedness with other DBD, and with the continuum of “normal” human behavior, has direct implications for pedigree interpretation, variant analysis, and risk assessment.

## Genetic Evaluation of ASD

Children with autism and other neurodevelopmental concerns represent a significant percentage of referrals for clinical genetics evaluation, with etiological investigation being the primary indication. As in other areas of medical practice, genetic testing for individuals with ASD has moved beyond the purview of clinical genetics and is being ordered by developmental pediatricians, neurologists, psychiatrists, and other specialists, as well as by primary care providers. Pediatric genetic counselors therefore see children with ASD referred through a variety of portals and having undergone various degrees of genetic diagnostic work-up. Prenatal genetic counselors routinely field inquiries about ASD recurrence from expectant couples with a previous affected child, or more commonly, a family history of ASD. Within their growing specialty area, psychiatric genetic counselors increasingly encounter adults with mental illness who report autism symptoms in themselves and/or other family members. Additionally, a growing army of research and laboratory-based genetic counselors is employed on the front lines of variant interpretation for genes implicated in ASD. Thus, the impact of the theoretical “sea change” about ASD’s connections to other neurodevelopmental disorders, and to adult-onset psychiatric conditions, has direct relevance across several different areas of genetic counseling practice.

Prior to the advent of array and whole genome/exome sequencing technologies, genetic contributors to ASD were largely unknown, and genetic counseling focused on empiric recurrence risks for ASD, occasionally informed by a positive pedigree. Consensus guidelines from national professional organizations, including the American College of Medical Genetics, the National Society of Genetic Counselors, and the American Academy of Pediatrics, recommend consideration of fragile X and CMA for children diagnosed with ASD [38–41], and increasingly, clinical WES is being ordered as a standard part of the etiological evaluation of neurodevelopmental disorders. Although specific genetic causes are individually rare in ASD, they collectively represent its most significant known etiology [8, 15•, 28, 42]. Whole genome CMA reveals a pathogenic CNV in 15–20 % of individuals with unexplained developmental delay, ID, ASD, or multiple congenital anomalies [43, 44]. The reported yield is 7–14 % in studies restricted to the evaluation of individuals with ASD [45–50]. Large laboratory-based clinical WES studies have consistently identified a pathogenic SNV in 26–29 % of people with neurodevelopmental disorders in general [51–53], including 8–20 % of those with ASD [50, 52, 53]. Among children with ASD who underwent both CMA and WES testing, the combined molecular diagnostic yield was 15.8 % [50].

When a specific cause can be identified for ASD, genetic counseling has traditionally followed familiar processes with regard to risk assessment, explanations of inheritance, recommended family testing, anticipatory medical guidance, and discussion of psychosocial aspects of genetic disorders. For well-known genetic causes of ASD, such as fragile X syndrome, there may be an abundance of resources and established support organizations for the family. More commonly, however, little is known about the genetic diagnosis, as is the case for the large number of newly identified rare, pathogenic CNVs and SNVs implicated in autism. These include pathogenic microdeletions of 15q11.2 and loss of function variants in *NRXN1* that can cause ASD but also confer risk for a wide range of other neurodevelopmental and psychiatric diagnoses, from ID to SCZ, and are found as well in seemingly unaffected individuals [34, 36, 50, 51, 54]. With few exceptions, the phenotypic effects of this recent generation of CMA and WES-detected etiologies of autism are nonsyndromic, meaning that they do not induce structural organ defects, overt dysmorphic features, or significant medical comorbidities. For these newly identified causes, genetic counseling relies heavily on variant analysis and pedigree interpretation and requires a broader discussion of neurodevelopmental and psychiatric risk beyond a family’s focused concern about autism. This represents a marked departure from the traditional model of genetic counseling in which ASD is described as one specific characteristic among a syndrome’s physical and medical features [25•].

## The DBD Pedigree

A hallmark of genetic counseling practice is the ability to construct a detailed family pedigree in order to inform genetic risk assessment. When a consistent physical trait is present, as in families with multigenerational cystic kidneys, for example, a pedigree serves as a visual shorthand that allows the genetic counselor to quickly deduce an inheritance pattern. For neurodevelopmental and psychiatric phenotypes, these patterns are not quite so straightforward and often obscured by artificial diagnostic distinctions, masking the true magnitude of DBD recurrence. In medical genetics, ASD, EP, BPD, cerebral palsy, and other DBD are still widely and incorrectly viewed as unrelated conditions, each one designated with its own distinct symbol or colored quadrant in a pedigree to emphasize their presumed lack of connection to each another [25•]. Likewise, recurrence estimates for ASD have historically been based on single-minded analyses of repeated instances of ASD (vs. no ASD) in families, despite the eagerness of parents to point out an uncle with obsessive compulsive disorder or a sibling with significant language impairment. Once dismissed as irrelevant, the importance of these seemingly unrelated diagnoses in other relatives is now being appreciated and forcing a reexamination of the long-held genetic tenets of nonpenetrance and variable expressivity, at least in terms of neuropsychiatric phenotypes [24•, 25•, 54].

Studies published in the last decade suggest that the empiric risk for recurrence of idiopathic ASD hovers around 10 % (range ~7 to 14 %) for couples with one affected child [55–59, 60•], but may be as high as 32–36 % for couples who already have two or more children with idiopathic ASD [57, 61]. Only recently have studies begun to connect the dots between autism and different types of DBD, and the recurrence risk for *any type of DBD* in families with one ASD proband is far higher than previously imagined [60•]. For example, in a large Finnish epidemiologic study involving thousands of families, the prevalence of ASD among siblings of probands with ASD was 10.5 %, but almost 37 % of these siblings had some type of neurodevelopmental or psychiatric disorder (vs. 17.4 % of controls) [60•]. Specifically, the risk among siblings was significantly increased for tic disorder, ADHD, ID, learning or coordination disorder, conduct or oppositional disorder, childhood-onset emotional disorder, SCZ spectrum disorder, affective disorder, and anxiety disorder [60•]. Recurrence risks for siblings were similar whether or not the proband had ID and irrespective of proband gender. Much smaller studies have suggested that 20–25 % of siblings who do not meet criteria for ASD have a history of language impairment or delay [56, 62]. It is increasingly apparent that the traditional pedigree designating ASD, ID, and other DBD as distinct and unrelated conditions in a family is fundamentally flawed. Likewise, recurrence risk estimates that address only the isolated chance for ASD without referencing the significantly higher chance for other DBD can no longer be considered acceptable.

Families who seek genetic counseling about autism are generally unaware of its newly discovered cross-connections with other DBD. Traditionally, genetic counselors list ASD, along with a condition’s other known physical and behavioral traits, and cite its relative chance of occurring as part of the disorder [25•]. While a syndrome’s physical manifestations can be accurately described in an all-or-none, categorical way (e.g., 75 with vs. 25 % without a congenital cardiac defect), the same is not true of behavioral and cognitive symptoms. For example, approximately 15 % of children with a 22q11.2 deletion meet behavioral criteria for an ASD diagnosis [63]. A parent might easily assume that 85 % of those with a 22q11.2 deletion are completely unaffected by ASD, not appreciating that autism symptoms occur along a continuum that extends beyond the black and white cutoff for a clinical ASD diagnosis. Describing the prevalence of a syndrome’s neurodevelopmental and psychiatric diagnoses in the same breath as congenital anomalies is misleading and fails to convey the continuously distributed nature of brain-based symptoms. Whether the etiology is unknown, due to a well-defined syndrome, or associated with a poorly understood genomic variant, the recognition that ASD is etiologically tethered to a host of other brain disorders and to the continuum of “normal” human behavior is forcing a reexamination of genetic counseling approaches. So far, clinical genetics professionals have paid relatively little attention to the seismic changes occurring in the fields of psychiatry and developmental medicine that will ultimately have a major impact on how autism and other brain disorders are defined, described, and treated [5, 24•, 26, 64–66]. “Reinventing” genetic counseling practices related to these disorders will arguably be one of the most important challenges facing the profession over the coming decade.

## Conclusions

Psychiatry is steadily moving toward a new conceptualization of brain disorders that blurs long-held diagnostic distinctions among neurodevelopmental and psychiatric conditions, including autism. Genomic discoveries lie at the heart of these changing perceptions, yet there has so far been minimal impact on traditional genetic counseling practices that continue to view ASD through the lens of a categorical, all-or-none risk model. Going forward, the genetic counseling field will need to significantly adapt its approaches to pedigree interpretation, variant analysis, and patient education to more precisely describe both the chance and the nature of autism recurrence in terms of a broader DBD continuum. These efforts will have implications for multiple practice areas and require philosophical changes for experienced practitioners and for the training of new genetic counselors. Resetting entrenched dichotomous notions about autism, ID, and other brain-based manifestations of genetic conditions will require a strategic educational effort on the part of the genetic counseling profession. Once accomplished, families seeking genetic counseling will benefit from a more accurate and contextual understanding of these disorders on which to base informed decisions.
